# ID 130 - Lei n.º 14.313/2022: uma alternativa regulamentar para integralidade do cuidado à população pediátrica no SUS?

**DOI:** 10.5327/2237-9622.2025.v34s1.130

**Published:** 2025-11-25

**Authors:** Andréa da Silva Dourado, Ana Carolina Scalize Dias, Anna Carolina dos Santos Ramalho, Isabella Mota dos Santos, Gilson Takazumi Higashi Lo, Daniela Oliveira de Melo

**Keywords:** protocolos clínicos, acesso a medicamentos essenciais, uso *off-labe*l, saúde da criança, Avaliação de Tecnologias em Saúde

## Abstract

**Introdução:**

A Lei n.º 14.313, de 21 de março de 2022, regulamentou a dispensação, no âmbito do Sistema Único de Saúde (SUS), de medicamentos com indicação de uso distinta da aprovada em bula, sem necessidade de autorização de uso concedida pela Agência Nacional de Vigilância Sanitária (Anvisa), quando houver recomendação pela Comissão Nacional de Incorporação de Tecnologias no Sistema Único de Saúde. Apesar de a dispensação desses medicamentos ter sido recentemente regulamentada, não é conhecida a situação de recomendação em Protocolos Clínicos e Diretrizes Terapêuticas (PCDT) – documentos normativos que estabelecem critérios de cuidado no âmbito do SUS. Assim, o objetivo do estudo foi identificar recomendações farmacológicas previstas em caráter em PCDT em relação à faixa etária, com ênfase na população pediátrica.

**Método:**

Foi realizada busca por PCDT nos sites oficiais do governo federal, sendo incluídos documentos com portarias publicadas até 3 de novembro de 2022 e que possuíssem lista formal de recomendação de medicamentos. Foram extraídas informações do PCDT, identificação dos medicamentos e das apresentações recomendados e faixa etária de indicação de uso. Para todas as especialidades farmacêuticas recomendadas, foi verificada a situação regulatória por meio da Plataforma Consultas Anvisa, com identificação da bula. Houve comparação da indicação de uso recomendada em PCDT e a prevista em bula. Foi definida como população pediátrica indivíduos com idade menor que 18 anos. Foi classificada como recomendação aquela cuja população indicada em PCDT não estava coberta pela faixa etária preconizada em bula.

**Resultados:**

Foram identificados 101 PCDT, contendo 1.020 especialidades farmacêuticas, das quais 428 (47,96%) foram recomendadas em caráter . A maioria das especialidades (n=278; 94,95%) estava registrada apenas para utilização na população adulta acima de 18 anos. Para o restante (n=150; 35,05%), a bula indicava a população adulta e pediátrica, porém restringia idade mínima para utilização do medicamento entre 6 meses e 16 anos.

**Conclusão:**

Aproximadamente metade das especialidades farmacêuticas previstas em PCDT era recomendada em caráter em relação à faixa etária indicada para utilização. O desafio no atendimento integral às necessidades da população pediátrica, observado na prática clínica, é refletido no número de especialidades farmacêuticas com restrição de idade em bula – fenômeno explicado pelas dificuldades na operacionalização de estudos que comprovem segurança e eficácia de medicamentos para essa população, demandando mais recursos e expertise. Observa-se uma reação em cadeia que parte da falta de estudos clínicos, percorre a ausência de registro sanitário de medicamentos e, por fim, é constatada no sistema de saúde na forma de lacunas assistenciais. A opção pela redução de restrições mais rigorosas em PCDT, o que inclui a faixa etária, pode ser uma tentativa de diminuir as dificuldades de acesso a medicamentos. Nesse sentido, alterações promovidas pela Lei n.º 14.313/2022 foram importantes para viabilizar a disponibilização de medicamentos para populações usualmente desassistidas, como a população pediátrica.

